# Novel norovirus recombinants detected in South Africa

**DOI:** 10.1186/1743-422X-11-168

**Published:** 2014-09-17

**Authors:** Janet Mans, Tanya Y Murray, Maureen B Taylor

**Affiliations:** Department of Medical Virology, Faculty of Health Sciences, University of Pretoria, Pretoria, South Africa

**Keywords:** Intergenotype, Intragenotype, Norovirus recombinants, Novel, South Africa

## Abstract

**Background:**

Noroviruses (NoV) are the leading cause of viral gastroenteritis worldwide. Recombination frequently occurs within and between NoV genotypes and recombinants have been implicated in sporadic cases, outbreaks and pandemics of NoV. There is a lack of data on NoV recombinants in Africa and therefore their presence and diversity was investigated in South Africa (SA).

**Results:**

Between 2010 and 2013, eleven types of NoV recombinants were identified in SA. Amplification of the polymerase/capsid region spanning the ORF1/2 junction and phylogenetic analysis confirmed each of the recombinant types. SimPlot and maximum *x*^2^ analysis indicated that all recombinants had a breakpoint in the region of the ORF1/2 junction (P < 0.05). The majority (9/11) were intergenotype recombinants, but two intragenotype GII.4 recombinants were characterised. Three combinations represent novel recombinants namely GII.P not assigned (NA)/GII.3, GII.P4 New Orleans 2009/GII.4 NA and GII.P16/GII.17. Several widely reported recombinants were identified and included GII.P21/GII.2, GII.P21/GII.3, GII.Pe/GII.4 Sydney 2012, and GII.Pg/GII.12. Other recombinants that were identified were GII.Pg/GII.1, GII.Pe/GII.4 Osaka 2007, GII.P4 New Orleans 2009/GII.4 Sydney 2012, GII.P7/GII.6. To date these recombinant types all have a reportedly restricted geographic distribution. This is the first report of the GII.P4 New Orleans 2009/GII.4 Sydney 2012 recombinant in Africa.

**Conclusions:**

Over the past four years, remarkably diverse NoV recombinants have been circulating in SA. Pandemic strains such as the GII.Pe/GII.4 Sydney 2012 recombinant co-circulated with novel and emerging recombinant strains. Combined polymerase- and capsid-based NoV genotyping is essential to determine the true diversity and global prevalence of these viruses.

## Background

Noroviruses (NoVs) are the leading cause of viral gastroenteritis worldwide. In recent years, the importance of NoV recombinants as gastroenteritis pathogens has increasingly been recognised. Norovirus has a single-stranded positive sense RNA genome, ranging between 7.3 and 7.7 kb [1]. The genome is divided into three open reading frames (ORFs), with ORF1 encoding a large polyprotein which is processed into six non-structural proteins. The major and minor capsid proteins are encoded by ORFs 2 and 3, respectively. Noroviruses are classified into 6 genogroups (G) of which GI, GII and GIV infect humans [1]. Currently, based on complete capsid gene sequences, the genogroups are further divided into nine GI, 22 GII and two GIV genotypes [1–3]. Routinely NoVs are genotyped by analysis of partial capsid or polymerase gene sequences. However, genotyping based solely on one region of the NoV genome is not a true representation of the epidemiology of the virus due to frequent recombination within NoVs [4]. Recombination commonly occurs at the ORF1-ORF2 junction [4] although other recombination sites have been reported [5, 6]. To address the inconsistencies in NoV genotyping, a new nomenclature system, which incorporates both polymerase and capsid regions has recently been proposed [7].

Several globally prevalent NoV strains have been characterised as recombinants. The current predominant GII.4 variant, Sydney 2012, is a recombinant between GII.Pe in the polymerase and GII.4 in the capsid region [8]. Another GII.4 recombinant between the New Orleans 2009 polymerase and Sydney 2012 capsid regions is also in circulation [9]. After GII.4, the GII.P21/GII.3 recombinant is the second most prevalent NoV strain detected in children with gastroenteritis [10]. Other recently described recombinants include the GII.Pg/GII.1 and GII.Pg/GII.12 strains [11, 12]. Since 2010, the GII.Pg/GII.1 recombinant has been associated with foodborne outbreaks of gastroenteritis in Belgium and Germany [11, 13]. Norovirus recombinant GII.Pg/GII.12 emerged in Australia in 2008 [14] and has since been reported in the United States (US), where it was responsible for 16% of gastroenteritis outbreaks in the 2009–2010 winter season [12], and in children in Italy during the same time period [15].

In South Africa (SA), NoV-associated gastroenteritis outbreaks were first reported in 1993, where Norwalk (GI.1) and Hawaii (GII.1) strains were each identified as causative agents in outbreaks [16]. In 2008, NoVs were characterised from paediatric patients hospitalised with gastroenteritis in the Pretoria region of SA. Noroviruses were detected in 14% of stool specimens and the characterised strains included three GI and eight GII genotypes with GII.4 being predominant [17]. These genotypes were determined based only on capsid gene sequences. Increased awareness of the circulation and emergence of NoV recombinants highlights the need to genotype based on both polymerase and capsid gene regions. In this study, the presence of known and novel recombinant NoVs is reported for the first time in SA.

## Results

**Figure 1:**
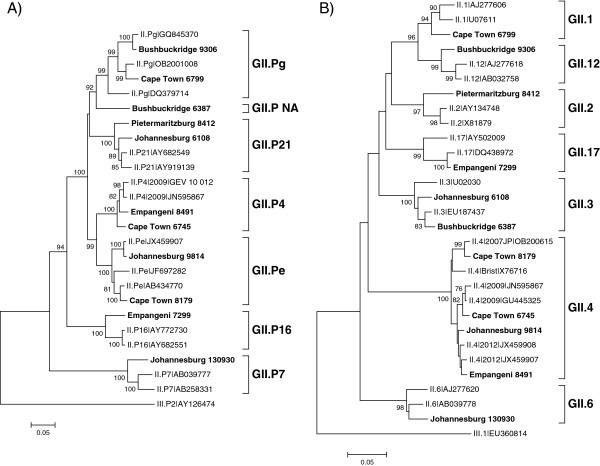
**Nucleotide phylogenetic analysis of A) the partial polymerase gene and B) the partial capsid gene of recombinant noroviruses (NoVs) detected in South Africa.** Neighbor-joining phylogenetic analysis of the partial polymerase (720 bp) region and the partial capsid (270 bp) region of 11 NoV recombinant strains. The NoV reference strains are indicated by GenBank accession numbers and the strains identified in this study are shown in bold. Statistical significance was evaluated with a 1000 bootstrap replicates and bootstrap support of >70% is indicated. The scale bar represents nucleotide substitutions per site.

**Figure 2:**
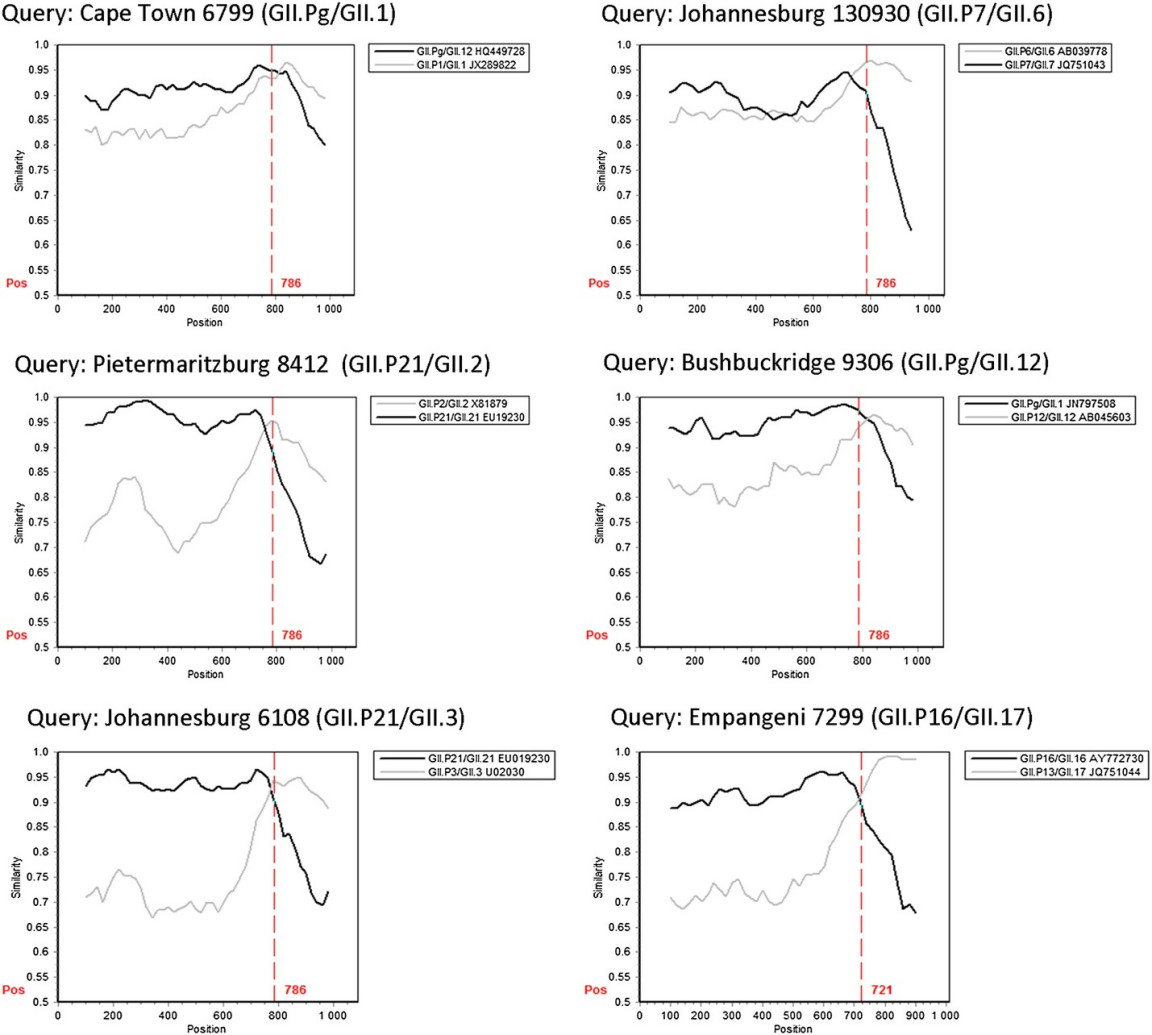
**Similarity plots of intergenotype NoV recombinants identified in South Africa.** The similarity plots were created with the polymerase/capsid sequence (1010–1090 bp) spanning the ORF1/ORF2 junction using SimPlot version 3.5.1, with a window size of 200 bp and an increment of 20 bp. In each graph, the black and grey lines represent the percentage identity of the putative parental strains to each respective recombinant. The start of ORF2 is indicated by the dashed red line (position 721 or 786). The predicted recombination breakpoint is where the parental strains share equal identity to the recombinant strain.

**Figure 3:**
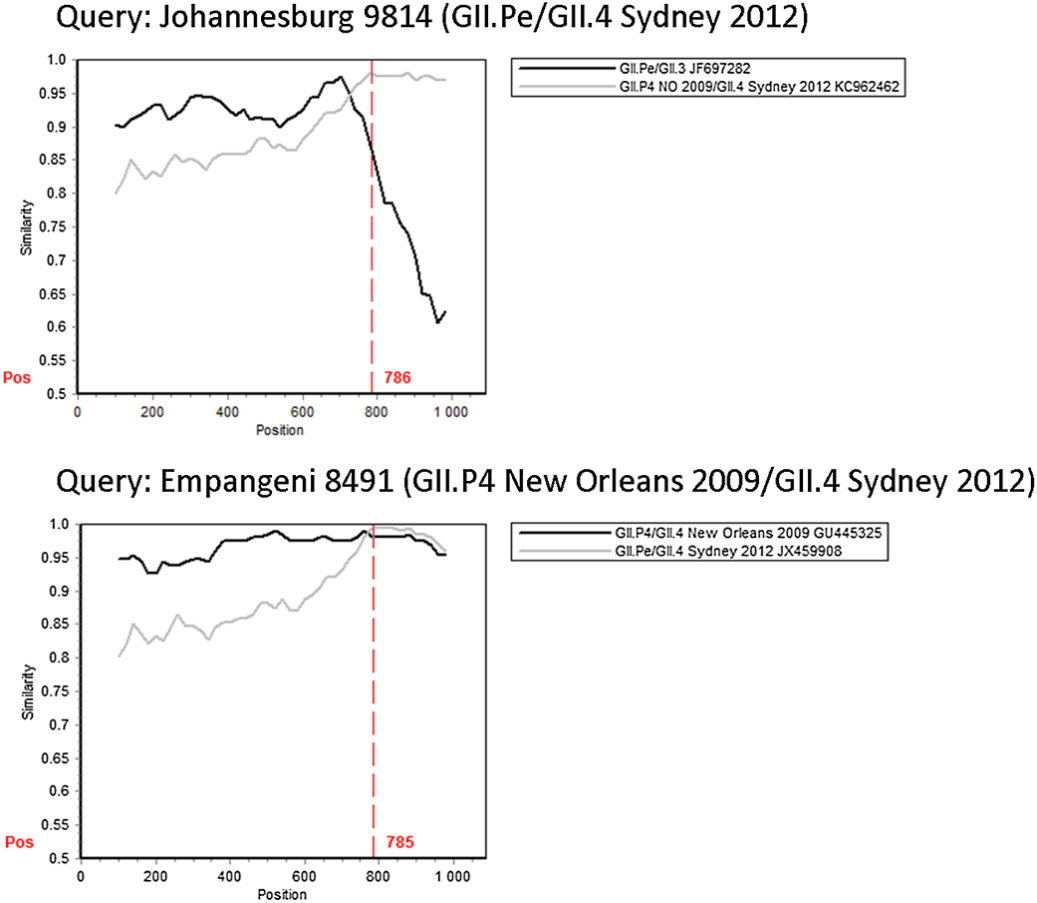
**Similarity plots of NoV GII.4 recombinants identified in South Africa.** The similarity plots were created with the polymerase/capsid sequence (1088 bp) spanning the ORF1/ORF2 junction using SimPlot version 3.5.1, with a window size of 200 bp and an increment of 20 bp. In each graph, the black and grey lines represent the percentage identity of the putative parental strains to each respective recombinant. The start of ORF2 is indicated by the dashed red line (position 785 or 786). The predicted recombination breakpoint is where the parental strains share equal identity to the recombinant strain.

From 2010 to 2013, eleven NoV recombinant types were identified in SA (Table 1). Ten of these recombinants were identified in children with NoV gastroenteritis and one recombinant originated from an adult with sporadic gastroenteritis. Phylogenetic analysis grouped the partial polymerase and capsid regions of each strain into different genotypes, suggesting recombination (Figure 1A and B). Subsequent maximum *x*^2^ and SimPlot analysis of a 1090 bp region, spanning the polymerase and capsid typing regions, indicated that all recombination breakpoints (P < 0.05) were at the ORF1/2 junction (Table 1, Figures 2 and 3). The majority were intergenotype recombinants and two GII.4 intragenotype recombinants were also characterised (Table 1, Figure 1).

**Table 1:** **Phylogenetic relationships and breakpoint location of recombinant norovirus strains circulating in South Africa from 2010–2013**

Year of detection	Strain name/GenBank accession number	Polymerase genotype	Capsid genotype	Breakpoint: SimPlot/***x*** ^2^	Estimated P-value (***x*** ^2^)	GenBank accession number of most closely related NoV strain (%identity/%coverage) ^#^	Worldwide detection ^#^
2010	Johannesburg 6108 KC962457	GII.P21	GII.3	−16/+56*	2.154 × 10^−13^	JX439787 (98%/99%)^a^	China, India, Korea
2010	Bushbuckridge 6387 KC962458	GII.P NA	GII.3	ND	ND	KC597144 (89%/99%)^b^	Novel polymerase region
2011	Cape Town 6745 KJ710245	GII.P4 New Orleans 2009	GII.4 NA	ND	ND	JX448566 (96%/99%)^c^	Novel capsid region
2011	Cape Town 6799 KC962459	GII.Pg	GII.1	−27/-88	1.608 × 10^−4^	JN797508 (93%/99%)^a^	Europe, US
2011	Empangeni 7299 KC962460	GII.P16	GII.17	−6/+17	1.610 × 10^−15^	JX683114 (93%/99%)^a^	Novel recombinant
2011	Cape Town 8179 KM025143	GII.Pe	GII.4 Osaka 2007	ND	ND	GQ845369 (97%/99%)^c^	Australia, India, Japan, US
2011	Pietermaritzburg 8412 KC962461	GII.P21	GII.2	−29/+14	1.846 × 10^−13^	AY682549 (96%/97%)^a^	France
2012	Empangeni 8491 KC962462	GII.P4 New Orleans 2009	GII.4 Sydney 2012	−15/-61	1.767 × 10^−3^	KF509947 (96%/99%)^c^	Australia, Canada, Europe, Asia, US
2012	Bushbuckridge 9306 KJ710246	GII.Pg	GII.12	−29/+68	9.441 × 10^−9^	JQ613568 (98%/99%)^a^	Asia, Australia, Europe, US
2012	Johannesburg 9814 KJ710247	GII.Pe	GII.4 Sydney 2012	−59/+20	8.515 × 10^−15^	KF145148 (98%/99%)^c^	Worldwide
2013	Johannesburg 130930 KJ710248	GII.P7	GII.6	−54/-36	8.238 × 10^−10^	KJ407072 (96%/98%)^a^	Japan, US

*Breakpoint position relative to the start of ORF2, NA – Not assigned, ND – Not determined, ^#^Based on BLAST analysis of ^a^the polymerase-capsid overlap region (~1090 bp); ^b^the complete polymerase-capsid overlap region (~1820 bp); and ^c^the partial polymerase-complete capsid region (~2429 bp).

**Figure 4:**
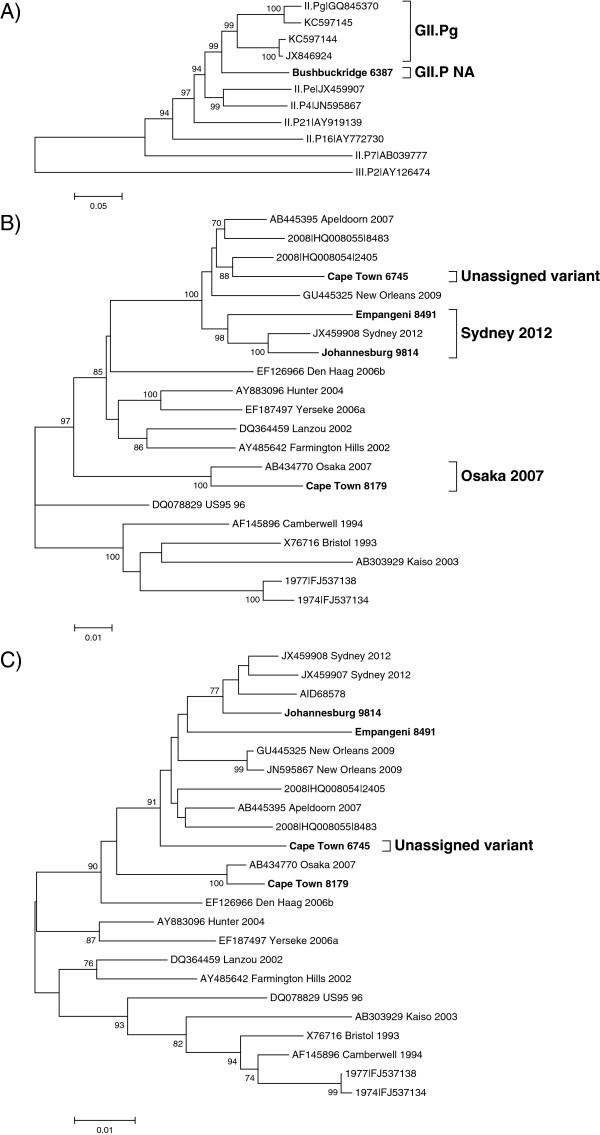
**Phylogenetic analysis of unassigned norovirus (NoV) recombinants.** Neighbor-joining phylogenetic analysis of **A)** the complete polymerase gene (1533 bp) of Bushbuckridge 6387 (GII.P NA/GII.3), **B)** the complete capsid (1623 bp) gene of NoV GII.4 variants and, **C)** the 540 amino acid sequence encoded by the capsid gene of the NoV GII.4 variants. For the amino acid tree the evolutionary distances were computed using the Poisson correction method and are in the units of the number of amino acid substitutions per site. The NoV reference strains are indicated by GenBank accession numbers as well as GII.4 variant names and the strains identified in this study are shown in bold. Statistical significance was evaluated with a 1000 bootstrap replicates and bootstrap support of >70% is indicated. The scale bar represents nucleotide or amino acid substitutions per site.

Three of the recombinants, GII.P not assigned (NA)/GII.3, GII.P4 New Orleans 2009/GII.4 NA and GII.P16/GII.17, are reported for the first time. Phylogenetic analysis of a 720 bp region of the polymerase gene of Bushbuckridge 6387 (GII.P NA/GII.3) did not group this strain with an established polymerase-based genotype, although the strain is most closely related to the GII.Pg strains (Figure 1A). Following nucleotide sequence determination of the complete polymerase gene it remained unassigned (Figure 4A). The closest matches on GenBank are GII.Pg/GII.3 (KC597144) and GII.Pa/GII.3 (JX846924) recombinants from China with which it shares 90% sequence identity. Cape Town 6745 is an intragenotype GII.4 recombinant with a New Orleans 2009 polymerase and an unassigned capsid region. The variant could not be assigned using the nucleotide or amino acid sequence of the complete capsid (Figure 4B and C). This strain is related to the Apeldoorn 2007 variant and shares 96% nucleotide identity with five strains in GenBank. The third novel recombinant is a combination of GII.P16 and GII.17. The most closely related strain on GenBank, a GII.P16/GII.3 recombinant detected in Bangladesh in 2012 (JX683114), is 93% identical to the SA strain.Strains with the GII.P21 polymerase were found in combination with GII.2 or GII.3 capsids, both of which are widely reported recombinants. The GII.P21/GII.2 and the GII.P21/GII.3 SA strains share 96% (AY682549) and 98% (JX439787) nucleotide identity with their respective closest matches from GenBank. The GII.Pg polymerase genotype was identified as a recombinant with GII.1 or GII.12 capsids. The GII.Pg polymerase sequences cluster in two distinct groups within the GII.Pg genotype (Figure 1A), with the GII.Pg/GII.1 sharing only 93% nucleotide sequence identity with the closest match in GenBank (JN797508). A recently emerged recombinant, GII.P7/GII.6, was detected in Johannesburg in 2013. The most closely related strain in GenBank is 96% identical over 98% of the polymerase/capsid overlap region and was identified in the US in 2010 (KJ407072).

In addition to the novel GII.P4 New Orleans 2009/GII.4 NA recombinant, three other GII.4 recombinants were identified. The currently globally predominant variant capsid, Sydney 2012, was found in combination with both the GII.Pe and GII.P4 New Orleans 2009 polymerase genotypes. The GII.Pe/GII.4 Sydney 2012 SA strain is 98% identical to strains circulating in Asia, Canada, Italy and Australia. The GII.P4 New Orleans 2009/GII.4 Sydney 2012 recombinant is most closely related (96% nucleotide sequence identity) to a strain from Canada (KF509947). A non-epidemic GII.4 variant, the GII.Pe/GII.4 Osaka 2007 recombinant was also characterised.

## Discussion

Combined characterisation of the polymerase and capsid regions of NoVs has increased awareness and detection of NoV recombinants worldwide. Consequently, it has become clear that NoV genetic diversity is greatly enhanced by inter- and intragenotype recombination.

This is the first report of NoV recombinants circulating in individuals with gastroenteritis in SA. One novel recombinant, Bushbuckridge 6387, with an unassigned polymerase region combined with a GII.3 capsid, shares 88% nucleotide identity over the polymerase region with two NoV strains that had circulated in Hong Kong, China between December 1977 and January 1978. The most recently described strain related to Bushbuckridge 6387 in the polymerase region is strain KC597145, a GII.Pg/GII.12 recombinant detected in 2010 in the US (86% nucleotide sequence identity). This reflects the evolutionary relationship between the unassigned polymerase and the GII.Pa and GII.Pg polymerases. The partial capsid sequence of this recombinant is 98% identical to a NoV GII.3 strain (AB385634) that was detected in 2005 in the Netherlands. It appears that a polymerase genotype that had been circulating undetected for many years has recombined with the prevalent GII.3 capsid genotype.

Another novel intergenotype recombinant detected in this study was the GII.P16/GII.17 strain. The GII.P16 polymerase was recently described in combination with the GII.2 capsid in China [18] and Japan [19] as well as with the GII.3 capsid in Bangladesh [20] and Italy [21]. In addition a GII.P16/GII.13 recombinant was also detected in children with gastroenteritis in Italy [21]. These data suggest that the GII.P16 polymerase could be predisposed to recombination. The GII.16 and GII.17 genotypes (based on the partial capsid gene) have been detected in SA in clinical specimens [17] and environmental samples [22, 23], respectively. Therefore mixed infections with GII.P16/GII.16 and GII.P17/GII.17 could have occurred providing the opportunity for recombination. Analysis of GII.P16 sequences available on GenBank revealed that this polymerase type is circulating in Africa (KJ394506), Asia (KJ145841) and Central America (KF177231) and that it has been detected on frozen strawberries implicated in a gastroenteritis outbreak in Germany (KC207117).

One of the most widely reported recombinant strains, GII.P21/GII.3, was also identified in this study. The SA strain is related to a subgroup of GII.P21/GII.3 recombinants, which have been reported in China [18], India [5] and Korea (JX439784-91). The GII.P21/GII.2 recombinant was first reported in 2005 [24] and the ORF1/2 overlap region of the SA strain is 96% identical to the prototype strain Pont de Roide 673/04/Fr. This intergenotype recombinant has not been reported as extensively. The GII.Pg/GII.1 has only been reported in Europe [11, 13] and the US (JN797508). The SA recombinant is not closely related (<93% nucleotide sequence identity) to any of the reported strains, specifically in the polymerase region. GII.Pg/GII.12 recombinants have been reported in Australia [14], China [25], Europe [15, 26] and the US [12, 27]. To date this is the first report of these recombinant types circulating in Africa.

One recombinant type recently described for the first time in Burkina Faso, GII.P7/GII.6, was also detected. The SA strain shares 94% identity over 98% of the nucleotide sequence from Burkina Faso [28], with only 92% nucleotide identity in the capsid region. The capsid region of the SA recombinant is most closely related (97% identity over 92% of Region C) to a Japanese GII.6 strain (AB919088) reported in Okinawa in 2014. This indicates that two lineages of GII.P7/GII.6 recombinants may be circulating in Africa. The closest match to the SA strain was a GII.P7/GII.6 from the US (KJ407072), with which it shares 96% nucleotide identity over 98% of the sequenced region. GII.P7/GII.6 recombinants have also been reported in Finland [26], Japan (AB818397-400) and Sweden (KF768487), although the strains from Finland remain unconfirmed.

Inter- and intragenotype GII.4 recombinants have been identified in SA. The GII.Pe/GII.4 Osaka 2007 variant, considered a non-epidemic variant, was detected in 2011. The SA variant is closely related (97% identity over 99% of the overlap region) to the original strain described in Japan in October 2007 (AB434770). Based on BLAST analysis, the SA GII.Pe/GII.4 Sydney 2012 recombinant is closely related to strains circulating on several continents [8, 29–31]. This is the first report of the globally dominant GII.Pe/GII.4 Sydney 2012 variant on the African continent. A GII.Pe/GII.4 recombinant has been reported from Burkina Faso [28] but the GII.4 variant is unassigned. Within the GII.4 lineage, recombination has been described between ORF1/2, ORF2/3 and within ORF2 [6]. In this study both intra- GII.4 recombinants had breakpoints at the ORF1/2 junction. The GII.P4 New Orleans 2009/GII.4 Sydney 2012 recombinant is less prevalent than the GII.Pe/GII.4 Sydney 2012 variant, with reports from Canada [32], Denmark [9] and Italy [33]. The presence of another intragenotype GII.4 recombinant in SA illustrates the remarkable diversity generated by recombination within the GII.4 genotype. The prevalent New Orleans 2009 polymerase appears to have recombined with an unassigned GII.4 capsid from the Apeldoorn 2007 lineage related to capsid genotypes which circulated in SA in 2008 [17]. In theory, the possibility cannot be excluded that these GII.4 intragenotype recombinants are the result of divergent evolution of the Apeldoorn 2007 lineage in the capsid gene. However, Eden and colleagues [6] have provided compelling evidence that recombination played an important role in the evolution of several recent GII.4 pandemic variants leading to an increase in the genetic repertoire of the major GII.4 lineage.

## Conclusions

Recombination contributes significantly to create diversity within NoVs. This study has revealed the presence of novel, emerging and widely reported recombinants in SA. The data presented here highlights the importance of combined polymerase- and capsid-based NoV genotyping to allow meaningful global epidemiological comparison of NoVs.

## Methods

### Specimen collection and preparation

From 2010 to 2012, NoV GII-positive stool specimens were selected for further genotypic characterisation. All specimens were received from the Rotavirus Sentinel Surveillance Programme which routinely screens stool specimens for enteric pathogens. Specimens were received from children up to the age of 5 years who were hospitalised with severe gastroenteritis in four provinces of SA: Gauteng (Johannesburg), KwaZulu-Natal (Empangeni and Pietermaritzburg), Mpumalanga (Bushbuckridge) and the Western Cape (Cape Town). In 2013, a NoV GII-positive stool specimen from a sporadic case of gastroenteritis in an adult was also characterised. Stool suspensions (10%) were prepared in ultrapure water (Adcock Ingram, Johannesburg, SA) and stored at −20°C until nucleic acid extraction.

### Nucleic acid extraction

For amplification of partial RNA polymerase (region A) and capsid (region C) gene regions, nucleic acid was extracted from 200 μl stool suspension using the MagNA Pure LC Total Nucleic Acid Isolation kit (Roche Diagnostics GmbH, Mannheim, Germany) on the automated MagNA Pure system (Roche Diagnostics). For amplification of a region spanning partial polymerase and capsid genes, nucleic acid was extracted from 140 μl stool suspension using the QIAamp Viral RNA Mini kit (Qiagen, Hilden, Germany). Nucleic acid was eluted in 50–60 μl and stored at −70°C until use.

### Genotyping of noroviruses – amplification and sequencing

Reverse transcription was performed using 10 μl extracted RNA and 50 U RevertAid™ Premium reverse transcriptase (Thermo Scientific, Waltham, MA), with 30 μM random hexamer primers. Region A (polymerase gene) was amplified using 5 μl of cDNA in a 50 μl reaction containing 200 μM dNTPs, 0.3 μM primers JV12Y and JV13I [34], 1.25 U AmpliTaq Gold DNA polymerase (Applied Biosystems, Foster City, CA) and 1.5 mM MgCl_2_. The reaction conditions were as follows: 95°C for 10 min, 40 cycles of 95°C for 30 sec, 37°C for 1 min 30 sec, 72°C for 1 min and a final step at 72°C for 5 min. Region C (capsid gene) was amplified using published primers G2SKF and G2SKR [35] as previously described [22]. The overlap region (1090 bp), spanning sections of the polymerase and capsid genes including the suspected recombination breakpoint, was amplified using primers JV12Y and G2SKR. Briefly, the 50 μl reaction contained 0.5 μl cDNA, 1.25 U AmpliTaq Gold DNA polymerase (Applied Biosystems), 200 μM dNTPs, 0.3 μM primer JV12Y and 1 μM primer G2SKR, using the following cycling parameters: 95°C for 10 min, 40 cycles of 94°C for 30 sec, 37°C for 1 min and 72°C for 2 min, followed by 72°C for 5 min. For strains that could not be genotyped based on partial polymerase or capsid sequences (remained unassigned phylogenetically), complete polymerase or capsid genes were amplified, respectively. For amplification of the complete polymerase gene, the following primer was designed: GIIpolF 5′-GTC ATC TGT GCA ACA CAA GG-3′ and used in conjunction with JV13I to amplify a 1038 bp region of ORF1. The 50 μl reaction contained 1.25 U AmpliTaq Gold DNA polymerase, 200 μM dNTPs, 0.5 μM of GIIpolF and 0.3 μM JV13I. The following cycling parameters were used: 95°C for 10 min, followed by 40 cycles of 95°C for 30 sec, 37°C for 1 min 30 sec and 72°C for 2 min, and a final extension at 72°C for 5 min. A consensus sequence was created using this amplicon and the overlap amplicon to obtain the complete RNA polymerase gene (1533 bp). A two-step semi-nested RT-PCR was used to amplify the 1623 bp capsid gene. In the first reaction, 1 μl of cDNA was combined with 1.25 U AmpliTaq Gold DNA polymerase, 200 μM dNTPs, primers QNIF2 [36] (200 nM) and GII.4 capsid reverse (5′-CCA TTA TAA WRC WCG YCT RCG CC-3′) (600 nM) and PCR buffer containing 1.5 mM MgCl_2_. One microliter of the first round PCR product was used as template in the second PCR, which used the same reaction mix except for forward primer G2SKF. The following cycling parameters were used: 95°C for 10 min, 35 cycles of 94°C for 45 sec, 58°C for 1 min and 72°C for 1 min 40 sec, and a final extension at 72°C for 10 min. All primers were manufactured by Applied Biosystems.

Amplicons from the partial RNA polymerase and capsid gene regions were sequenced directly with the ABI PRISM BigDye® Terminator v.3.1 Cycle Sequencing kit (Applied Biosystems) on an ABI 3130 automated analyser (Applied Biosystems). The overlap amplicon, partial ORF1 and complete capsid amplicons were cloned before sequencing, using the CloneJET™ PCR cloning kit (Thermo Scientific). At least two randomly selected clones were sequenced using pJET1.2/blunt specific primers (Thermo Scientific).

### Genotyping of noroviruses – phylogenetic analysis

Nucleotide sequences were edited and analysed using Sequencher™ 4.9 (Gene Codes Corporation, Ann Arbor, MI), BioEdit Sequence Alignment Editor (V.7.0.9.0) [37] and BLAST-n [38]. The polymerase and capsid genotypes were determined using the Norovirus Genotyping Tool [2] as well as phylogenetic analysis performed in MEGA6 [39]. Sequences were aligned with reference strains using MAFFT version 6 (http://mafft.cbrc.jp/alignment/server/index.html) and phylogenetic analysis was performed using the neighbor-joining method, validated by 1000 bootstrap replicates. Genotypes were assigned based on clustering with reference strains in the phylogenetic tree with >70% bootstrap support.

### Genotyping of noroviruses – analysis of recombination

Selected NoV strains that clustered with different genotypes based on phylogenetic analysis of the polymerase and capsid sequences were subjected to further recombination analysis. Putative recombination breakpoint analysis was performed using SimPlot version 3.5.1 and the maximum *x*^2^ test as implemented in RDP version 4.33 [40].

### Nucleotide sequence accession numbers

Sequences were submitted to GenBank under the following accession numbers: KC962457-62; KJ710245-48; KM025143.
